# Metatranscriptome profile of agricultural microbial communities enriched for plastitrophy

**DOI:** 10.1002/mlf2.70023

**Published:** 2025-07-28

**Authors:** Fatai A. Olabemiwo, Yuting Huang, Macy Thompson, Hanan Omar, Siddhant Kalra, Philip Arevalo, Valerie Nazzaro, Frederick M. Cohan

**Affiliations:** ^1^ Department of Biology Wesleyan University Middletown Connecticut USA; ^2^ College of Integrative Science Wesleyan University Middletown Connecticut USA; ^3^ Quantitative Analysis Center (QAC) Wesleyan University Middletown Connecticut USA

## Abstract

This study identified potential plastic‐degrading microorganisms and enzymes in agricultural soils using a novel two‐phase enrichment approach. By culturing agricultural soil in a Winogradsky column supplemented with polyethylene (PE) sheets, followed by culture in minimal medium with low‐density polyethylene (LDPE) microplastic, we identified 192 genes specifically upregulated in LDPE conditions, including 10 genes encoding known plastizymes and 182 genes encoding putative plastic‐degrading enzymes. Detailed enzyme classification revealed predominant roles for oxygenases (20%) and dehydrogenases (19%), with specific subclasses showing distinct distribution patterns. These findings expand our understanding of microbial responses to plastics in agricultural environments and provide a foundation for developing bioremediation strategies to address plastic contamination in soils.

The rapid increase in global plastic use has led to significant environmental and public health challenges, with an estimated 380 million tons of plastic waste generated annually and only 9% effectively recycled^1^. Agricultural soils are particularly vulnerable to plastic contamination due to various industrial practices, such as the application of sewage sludge and the use of plastic mulching^2^. Plastics in farmland contaminate farm produce with microplastics, providing a direct route for human ingestion^3^. Hence, there is an urgent need to reduce plastic pollution in agricultural environments.

Efforts to mitigate plastic pollution have been explored using bacteria and fungi that can partially degrade plastics into their safer monomer constituents. Bioremediation of plastics is possible because, although plastics are novel compounds, their chemical bonds are not. Many enzymes in nature have evolved to break down carbon‐based structures found in plastics, making it plausible that many microorganisms possess the ability to degrade plastics. The plastic enzyme databases^4^, ^5^ catalog numerous enzymes with plastic‐degrading capabilities, such as the highly efficient PETase from the bacterium *Ideonella sakaiensis* ^6^. Thus, the capacity to break down plastics in certain microbes likely arises from their inherent ability to degrade similar carbon‐based compounds found in nature. Previous studies have identified various soil bacteria capable of polyethylene (PE) degradation, particularly *Pseudomonas* species. Our study expands on this knowledge by first using a novel Winogradsky column approach to enrich and identify potential PE degraders and associated enzymes in agricultural soils, and then identifying enzymes whose expression is increased in plastic. Here, we focus on the critical issue of PE accumulation in agricultural soil due to increased use of PE mulching sheets^7^, ^8^.

Culture‐dependent techniques, such as growing environmental samples on plastic‐laced agar or exposing plastic strips in situ, have revealed various species with the ability to degrade plastics^9^, ^10^. To further explore the diversity of plastic‐degrading bacteria, here, we have added sheets of PE to a Winogradsky column. The Winogradsky column provides a continuum of natural conditions through layered incubation of samples. Over months, bacterial communities develop under oxygen, light, and chemical gradients within the column, allowing niche specialization, unlike culture on agar or through direct exposure to plastic in situ^11^, ^12^. We have previously shown that a Winogradsky column supplemented with PE sheets can increase the abundance of plastic‐degrading bacteria^12^.

In this study, we enriched and characterized plastic‐degrading bacteria and genes from agricultural soil in Connecticut, USA, on such a Winogradsky column. After 16 months of culture in the column, we focused on the bacteria growing on the biofilms associated with the PE sheets. From the biofilms, we screened for genes that were upregulated when cultured on minimal medium supplemented with low‐density polyethylene (LDPE) for 7 days. We thereby aimed to identify genes that were not previously known to degrade plastic but are nevertheless classified into broad enzyme categories associated with plastic metabolism.

We hypothesized a gene to be a potential plastic‐degrading gene if it belonged to one of the targeted functional categories in Table S1, and if, in transcriptome analyses, it showed a significant increase in abundance in plastic culture compared to a control culture lacking plastic. By concentrating on targeted functional categories, our study aimed to provide insights into microbial adaptations to plastic pollution and to identify key enzymes and metabolic capabilities that contribute to PE degradation in agricultural soils.

We considered the bacterial growth as biofilms on the PE sheets in the column as a possible first step in plastitroph enrichment, as our earlier work showed such biofilms to be enriched for plastitrophs and plastizymes^12^. However, we noted that this step may have alternatively enriched for ability to form a biofilm on a smooth substrate^13^. In any case, our next step was to use multi‐omics on the biofilm communities, to assay for upregulation of genes specifically associated with plastic degradation in the presence of LDPE. We focused on key metabolic pathways crucial for PE degradation: dehydrogenases, hydrolases, lipases, esterases, and hydroxylases (Table S1)^14^, ^15^, ^16^. We identified putative plastic‐degrading enzymes by comparing their expression in LDPE microplastics versus glucose conditions and cross‐referencing with known plastizyme functions.

Our findings span from visual examination (Figure S1) and taxonomic identification to the discovery of novel putative plastic‐degrading genes, providing insights into the genetic and enzymatic mechanisms underlying microbial plastic degradation in agricultural soils.

We characterized taxon diversity on the PE biofilms through 16S rRNA gene sequencing (Figure S2). For the metatranscriptomic analysis, we cultured bacteria from the PE strips in BH broth supplemented with either LDPE or glucose for 7 days (Figure S3).

Direct sampling of the PE biofilms from the Winogradsky columns revealed a high diversity of bacterial phyla at all levels of oxygenation. In the 16S rRNA survey, five of the top ten most‐abundant phyla included known plastitrophs: *Pseudomonadota* (14.5%), *Acidobacteriota* (14.2%), *Chloroflexota* (15.8%), *Bacteroidota* (7.8%), *Bacillota* (5.34%), and *Verrucomicrobiota* (2.82%). Another five of the top ten phyla are not yet known to degrade plastics, including *Planctomycetota* (11.9%), *Gemmatimonadota* (2.39%), *Crenarchaeota* (3.56%), *Desulfobacterota* (2.51%), and *Halobacterota* (2.59%) (Figure S2).

In contrast, enriching the biofilm bacteria in minimal media selected primarily for members of the *Pseudomonadota* and *Bacillota* phyla in our metatranscriptome, whether supplemented with LDPE or glucose. The phylum *Pseudomonadota* was more strongly enriched as a plastitroph than the *Bacillota*, with *Pseudomonadota* showing greater enrichment in LDPE (71.3% in glucose versus 83.1% in LDPE) and *Bacillota* showing greater enrichment in glucose culture (reaching 27% in glucose versus 13.5% in LDPE) (Figure 1A) (*p* < 2.2e−16).

**Figure 1:**
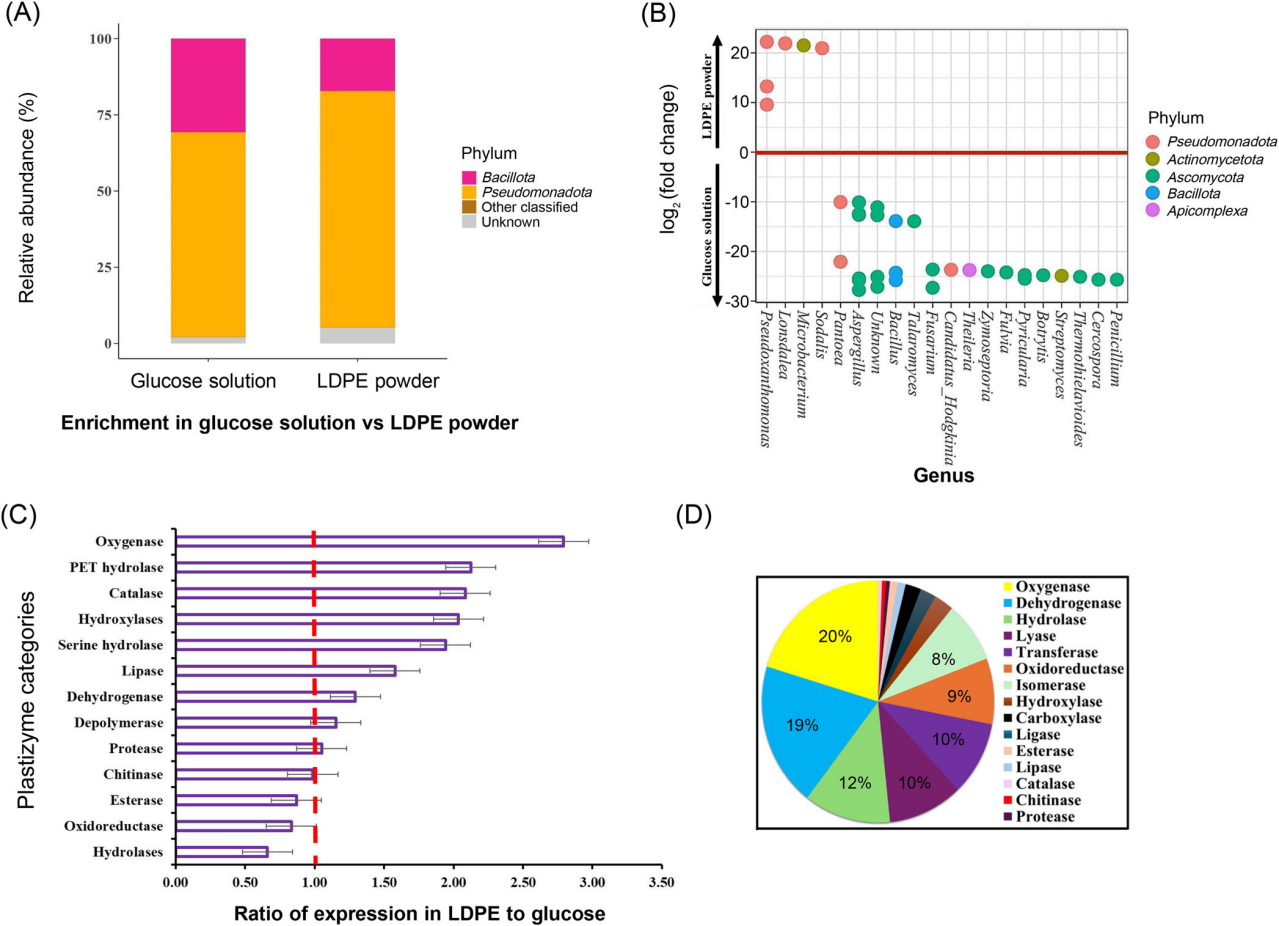
Microbial community dynamics and plastizyme profiles in low‐density polyethylene (LDPE) versus glucose conditions. (A) Relative abundance of dominant bacterial phyla (*Bacillota* and *Pseudomonadota*) and other classified microorganisms in minimal media supplemented with either glucose solution or LDPE powder. (B) log_2_(fold change) in abundance at the genus level between LDPE and glucose conditions, with taxa colored by their respective phyla, demonstrating significant shifts in community composition. (C) Expression ratios of plastizyme categories in LDPE relative to glucose conditions, with oxygenases, PET hydrolases, and catalases showing the highest upregulation. (D) Functional classification of putative plastizymes identified from LDPE‐enriched transcripts, revealing the predominance of oxidative and hydrolytic enzymes in LDPE degradation pathways.

We further explored this observation by examining the species whose transcriptomes were differentially enriched in LDPE versus glucose (Figure 1B). Five species belonging to various genera of *Pseudomonadota*, namely, *Pseudoxanthomonas* (3), *Lonsdalea* (1), and *Sodalis* (1), as well as one species of *Microbacterium* in the *Actinomycetota*, were significantly (*p* < 0.05 after Bonferroni adjustment) more enriched in LDPE than in glucose culture.

These results corroborate previous research that has identified *Pseudomonadota* as a phylum containing several known plastic degraders. For instance, a study by Danso et al.^17^ found that *Pseudomonas*, the type genus of *Pseudomonadota*, was one of the most abundant taxa in a microbial consortium capable of degrading polyethylene. Similarly, Jacquin et al.^18^ reported that *Pseudomonas* species were enriched in the presence of polyethylene and showed potential plastic‐degrading capabilities. Moreover, a review by Urbanek et al.^19^ highlighted the role for *Pseudomonas* in the biodegradation of various plastic types, including polyethylene, due to their ability to produce enzymes such as alkane hydroxylases and monooxygenases. These studies, along with our findings, underscore the importance of *Pseudomonadota*, particularly the genus *Pseudomonas*, as key players in the microbial degradation of LDPE and other plastics. Further research into the specific mechanisms and enzymes used by these microorganisms could lead to the development of more efficient plastic biodegradation strategies.

It is important to note that the subculture in flasks likely altered the bacterial community composition from that in the original Winogradsky column. While this step was necessary for our metatranscriptomic analysis, it may have selected for faster‐growing organisms adapted to the new conditions. Therefore, our gene expression results should be interpreted as representing the potential of the community under optimal growth conditions rather than directly reflecting in situ activities. We acknowledge that the submerged conditions in our Winogradsky column and subsequent flask cultures may have selected for organisms adapted to these specific conditions rather than reflecting the complete in situ soil community.

We applied a multi‐step approach to the 7184 genes/proteins of our metatranscriptome dataset to identify both known and putative plastizymes. Initial database matching with the Plastic Microbial Biodegradation Database (PMBD) and the Plastic Database (PDB) identified 51 known plastizymes (Figure 1C and Table S3). A paired *t*‐test demonstrated that these known plastizymes, as a set, were significantly more highly expressed in LDPE culture compared to glucose culture (*p* < 0.05). This indicates that LDPE as a sole carbon source induces the expression of genes previously associated with plastic degradation.

To identify novel, putative plastizymes, we first filtered for genes with substantial expression (abundance ≥ 50 TPM) in the pool of LDPE or glucose conditions, resulting in a refined set of 4969 transcripts (set N). We then divided this set into two subsets—genes with higher expression in LDPE than in glucose cultures (expression ratio ≥ 1.5, subset M, 1027 transcripts) and those with lower or similar expression (expression ratio ≤ 1.5, subset M′, 3942 transcripts). We recognized 10 genes in the LDPE‐upregulated subset M as known plastizymes, since they were found in the plastic databases (Table S3). Within the LDPE‐upregulated subset M, we identified 182 putative plastizymes (subset K), based on their greater expression in LDPE than in glucose and on their classification into the general functions related to plastitrophy (left column of Table S1). In the subset not enriched in LDPE (subset M′), we found 387 enzymes classified into the general functions related to plastitrophy (subset K′). Genes that were substantially more expressed in LDPE than in glucose were twice as likely to be classified into functional categories related to plastitrophy (odds ratio test and 2 × 2 contingency table, *p* < 0.0001).

Among the putative plastizymes in the LDPE‐enriched subset, we observed a notable distribution of enzyme classes associated with plastitrophy (Figure 1D). Oxygenases (20%) were the most abundant, followed by dehydrogenases (19%), hydrolases (12%), transferases (10%), and lyases (10%) (Figure 1D). The remaining categories included oxidoreductases (9%), isomerases (8%), hydroxylases (3%), ligases (2%), carboxylases (2%), esterases (1%), lipases (1%), catalases (1%), chitinases (1%), and proteases (1%). Within the oxygenase group, monooxygenases dominated (11% of total), with alkane and aromatic monooxygenases each comprising 4% of the total upregulated enzymes. The dehydrogenase group showed a balanced distribution between alcohol/aldehyde dehydrogenases (8% total), acyl‐CoA dehydrogenases (7% total), and other substrate‐specific dehydrogenases (4% total) (Table 1). This detailed classification reveals the specific enzyme subtypes involved in potential PE degradation pathways. This diverse enzymatic profile highlights the complex nature of LDPE biodegradation processes. Particularly, genes classified in the xenobiotic degradation (aromatic) category dominated the identified putative plastizymes expressed in the LDPE culture. This finding aligns with previous studies, such as Wierckx et al.^14^, who reported enrichment of genes involved in degrading aromatic compounds in microbial consortia capable of degrading polystyrene. We have listed the novel, putative plastizymes by the general functional category in Table S4.

**Table 1:** Classification and expression ratios of major upregulated enzymes in low‐density polyethylene (LDPE) degradation.

Class/subclass	% Total	Representative genes/enzymes	Expression ratio (LDPE/glucose)	Function
DEHYDROGENASES (46% total)				
1. Alcohol/Aldehyde				
a. Alcohol dehydrogenases	4	*yiaY, gcd*	6.09–1.74	Alcohol oxidation
b. Aldehyde dehydrogenases	4	*mmsA, feaB*	1.67–1.79	Aldehyde oxidation
2. Acyl‐CoA				
a. Long‐chain	2.5	ACADL	5.19	Long‐chain fatty acid oxidation
b. Medium‐chain	2.6	ACADM	2.19	Medium‐chain fatty acid oxidation
c. Short‐chain	1.9	Various	1.50–2.00	Short‐chain fatty acid oxidation
3. Substrate‐specific				
a. Malonate dehydrogenase	2	*mdcA*	2.83	Malonate metabolism
b. Citronellyl‐CoA	2	*atuD*	1.85	Terpene degradation
OXYGENASES (27% total)				
1. Monooxygenases				
a. Alkane	4	*ladA*	3.23	Alkane oxidation
b. Aromatic	4	*vanA, pobA*	1.86–1.58	Aromatic compound oxidation
c. Phenol/toluene	3	*dmpM, tmoF*	13.01–3.65	Phenolic compound oxidation
2. Dioxygenases				
a. Ring‐cleaving	3	*antA, pcaH*	4.58–1.53	Aromatic ring cleavage
b. Aromatic	3	*benA, xylX*	2.04	Aromatic ring hydroxylation
c. Protocol‐specific	3	*ligB*	2.06	Specific substrate oxidation

Expression ratios represent fold change in transcript abundance between LDPE and glucose conditions (LDPE/glucose). Only enzymes with significant upregulation (fold change >1.5, *p* < 0.05) are shown. Percentages in parentheses indicate the proportion of each enzyme class within total up‐regulated enzymes. Statistical significance was determined using DESeq2 with Benjamini–Hochberg correction.

Our results expand upon previous understanding of enzymatic processes in plastic degradation. While the earlier study emphasized the importance of hydrolases, depolymerases, and lipases in the initial polyethylene breakdown^20^, our findings suggest a potentially overlooked major role for dehydrogenases and oxygenases. These enzymes may be involved in subsequent steps of the degradation process, possibly in the oxidation of intermediates produced by initial polymer breakdown^17^, ^18^.

We also observed that genes related to cell growth, sporulation, quorum sensing, and competence development were highly enriched in the glucose culture but not in the LDPE culture (Table S5).

Interestingly, despite *Pseudomonas* being a well‐known plastic degrader, it was not prominent in our enrichments in either the Winogradsky column or minimal medium with LDPE. This could be due to competition from other organisms better adapted to our specific enrichment conditions. While our study captured the community composition after 16 months of enrichment, it is likely that the community underwent significant changes over this period, with initial dominance of fast‐growing, generalist species, followed by a shift toward more specialized, potentially PE‐degrading organisms.

Our metatranscriptomic analysis provides insights into the expression profile of known plastizymes and the identification of potential novel plastizymes under LDPE enrichment conditions. The combined approach of database matching, functional category analysis, and statistical testing can be effective in identifying both known and potential plastizymes. This methodology could be valuable for future studies aiming to discover novel plastic‐degrading enzymes in various microbial communities.

## AUTHOR CONTRIBUTIONS

**Fatai A. Olabemiwo**: Conceptualization; data curation; formal analysis; funding acquisition; investigation; methodology; supervision; validation; visualization; writing—original draft; writing—review and editing. **Yuting Huang**: Data curation; formal analysis; investigation; validation; writing—original draft; writing—review and editing. **Macy Thompson**: Data curation; formal analysis; investigation; validation; writing—original draft; writing—review and editing. **Hanan Omar**: Data curation; formal analysis; investigation; writing—review and editing. **Siddhant Kalra**: Formal analysis; methodology; writing—review and editing. **Philip Arevalo**: Conceptualization; formal analysis; writing—review and editing. **Valerie Nazzaro**: Formal analysis. **Frederick M. Cohan**: Conceptualization; formal analysis; funding acquisition; methodology; project administration; resources; supervision; validation; writing—original draft; writing—review and editing.

## ETHICS STATEMENT

This study involved soil sampling from Long Lane Farm at Wesleyan University, which was conducted with explicit permission from the farm coordinator. No human subjects, animal subjects, or endangered species were involved in this study. All sampling and experimental procedures complied with institutional and national guidelines for environmental research.

## CONFLICT OF INTERESTS

The authors declare no conflict of interests.

## Supporting information

Supplementary file‐13aMar2025.

TableS2.

TableS3.

TableS4.

TableS5.

## Data Availability

The raw sequencing data generated in this study have been deposited in the NCBI Sequence Read Archive (SRA) under the project numbers PRJNA1120435 (16S rRNA gene amplicon sequences) and PRJNA1120484 (metatranscriptomic sequences). All other relevant data are available within the manuscript and its Supporting Information files. Additional data or information related to this study are available from the corresponding author upon reasonable request.
