# Supplementary material for: Metatranscriptome profile of agricultural microbial communities enriched for plastitrophy
Source: mLife. 2025 Jul 28;4(4):465–9. doi: 10.1002/mlf2.70023 (PMC12396202; doi:10.1002/mlf2.70023)
Supplement: Supplementary file 1 — Supplementary file‐13aMar2025. [file MLF2-4-465-s005.docx]

Supplementary file for

**Metatranscriptome Profile of Agricultural Microbial Communities Enriched for Plastitrophy**

Fatai A. Olabemiwo ^1^, Yuting Huang ^1^, Macy Thompson ^1^, Hanan Omar ^1^, Siddhant Kalra ^1^, Philip Arevalo ^1,2^, Valerie Nazzaro ^3^, and Frederick M. Cohan ^1,*^

1. *Department of Biology, Wesleyan University, Middletown, Connecticut 06459, United States of America*
2. *College of Integrative Science, Wesleyan University, Middletown, Connecticut 06459, United States of America*
3. *Quantitative Analysis Center (QAC), Wesleyan University, Middletown, Connecticut 06459, United States of America*

*Corresponding author: Frederick M. Cohan [*fcohan@wesleyan.edu*](mailto:fcohan@wesleyan.edu)

**Materials and Methods**

*Soil collection and construction of a modified Winogradsky column*

We collected soil from Long Lane Farm, a student-run organic farm on the Wesleyan University campus. The site underwent environmental remediation where contaminated soil was removed and replaced with clean soil sourced from multiple uncontaminated locations in southern New England (1). The site's successful use for organic farming over the past decade demonstrates its restoration to agricultural quality. While comprehensive soil chemical analysis was not conducted for this study, the sustained crop production since remediation indicates a healthy soil environment suitable for studying agricultural microbial communities.

We collected two soil samples that were 5 meters apart (41°54'38"N, 72°66'69" W) on the farm on March 7, 2020, with permission from the farm coordinator. We used aseptic techniques to collect soil samples at a depth of 10 cm, and we pooled the samples.

We constructed a modified Winogradsky column by placing three pre-weighed sterile PE strips (3 x 2 cm, 40% crystallinity, and 0.1mm thickness) horizontally between four soil layers in a sterile 500 mL glass bottle, and we supplemented the column with Bushnell-Hass (BH) broth (2), an inorganic medium (MgSO_4_, CaCl_2_, KH_2_PO_4_, K_2_HPO_4_, FeCl_3_, and NH_4_NO_3_) to provide mineral nutrients for the microbes. A fourth plastic strip was placed in the liquid medium above the soil. We sealed the column with a 0.2 μm filter and incubated it at room temperature for 16 months.

*Collection and enrichment from biofilms on PE strips*

After sixteen months of incubation, we collected the PE strips from each layer of the Winogradsky column and detached the associated biofilm by vortexing in BH broth for 2 minutes. We focused on the second layer (aerobic-microaerophilic) of the Winogradsky column, as it represents a transition zone between aerobic and anaerobic conditions, similar to upper agricultural soil layers where plastic accumulation occurs. We inoculated biofilm samples from the second layer (aerobic-microaerophilic layer) into flasks containing 125 mL BH broth supplemented with either 50 g/L (0.28 M) glucose (Sigma aldrich, USA) or 50 mg low-density polyethylene (LDPE) powder (1-5 µM in size) (NanoChemZone, Canada). The switch to LDPE microplastics in this phase maximized surface area for potential microbial interactions. We incubated the cultures at 37°C with gyratory shaking at 200 rpm for 7 days, replicated in triplicate. After incubation, we retrieved samples from each flask through centrifugation to obtain cell pellets. Pellets were stored at -80°C with 200 uL of RNAlater reagent (RNAlaterTM, Thermo Fisher Scientific, Waltham, MA) for downstream metatranscriptomic analysis.

*DNA and RNA extraction and sequencing*

We extracted DNA from the detached biofilm samples using ZymoBIOMICS DNA isolation kits (Zymo Research Corporation, Irvine, CA, USA). The V4-V5 region of the 16S rRNA gene was amplified using 515F and 926R primers (3). Amplicons were sequenced on an Illumina MiSeq platform using 2x250 paired-end chemistry.

We extracted total RNA from the cell pellets of our 7-day liquid cultures using the Qiagen RNA Mini Prep Kit (QIAGEN, Germantown, MD, USA), followed by rRNA depletion and library preparation of community RNA. We assessed RNA quality and quantity on an Agilent 2100 bioanalyzer TapeStation and a Qubit 2.0 fluorometer. All samples had a DV200 value ≥ 50%, indicating sufficient RNA quality for generating metatranscriptomic libraries. We depleted 5S, 16S and 23S rRNA from 1 μg total RNA per sample using the QIAseq FastSelect -5S/16S/23S kit (QIAGEN, Germantown, MD, USA). We prepared strand-specific, directional libraries from the rRNA-depleted RNA using the NEBNext Ultra II Directional RNA library prep kit (New England BioLabs, Ipswich, MA, USA). We quantified the resulting libraries with a Qubit 2.0 Fluorometer, and pooled them in equimolar amounts for sequencing on an Illumina NovaSeq platform (Illumina, San Diego, CA) using 2 X 150 bp paired-end reads.

All sequencing was performed at Quintara Biosciences (Cambridge, MA), and raw reads were deposited in the NCBI Sequence Read Archive under project numbers PRJNA1120435 and PRJNA1120484.

*Taxonomic profiling of amplicon sequences*

From the Winogradsky column biofilms, 16S rRNA gene amplicon data were processed using a customized DADA2 v1.16 workflow (4) on the Nephele microbiome pipeline (5), including quality filtering, ASV inference, and taxonomic classification against the SILVA 16S rRNA database v138.1, retraining with RDP v18. We obtained 904,909 paired-end, raw Illumina reads from biofilms on the PE strips at the four levels of the oxygen gradient. The raw reads were subjected to a multi-step data processing pipeline to ensure high-quality input for downstream analysis. Initially, quality filtering was performed, yielding 876,962 valid reads. Subsequently, low-quality reads and chimeric sequences were removed, resulting in a high-quality set of 514,170 sequences. Applying DADA2 analysis (4), we identified 10,765 sequence variants (ASVs) across all samples. To minimize sequencing errors, we retained only ASVs present at ≥5 reads, resulting in a final set of 7,173 ASVs for characterizing the bacterial diversity inhabiting the Winogradsky’s PE biofilms.

*Assembly of metatranscriptome sequences*

From cultures on minimal medium supplemented with either LDPE or glucose, we assembled the metatranscriptome sequences through a multi-step process. First, we quality-filtered the reads using Fastp v0.23.2 (6) and removed ribosomal RNA using SortMeRNA v4.3.6 (7). We then processed the rRNA-filtered files using the WGSA2 pipeline on Nephele (5). For the de novo assembly, we employed metaSPAdes v3.15.2 (8), a specialized version of SPAdes designed for metagenome and metatranscriptome assembly. We set the identity threshold to 99% and used default parameters, which included iterative k-mer sizes of 21, 33, and 55. To ensure high-quality assemblies, we selected contigs with a minimum length of 500 base pairs for further analysis. Following the assembly, we aligned the reads using Bowtie2 and SAMtools. For taxonomic profiling, we utilized Kraken2 v2.0 (9).

We obtained a total of 56,197,433 paired-end reads from the pool of LDPE and glucose enrichments (Table S2). Quality filtering of the raw reads resulted in 56,086,521 unique, high-quality reads, which were assembled into 260,080 contigs. The contigs’ N_50_ values ranged from 7,127 to 15,619 across the samples. Following assembly, we generated a total of 324,385 transcripts across all samples, of which 7,185 transcripts were expressed in the LDPE and glucose enrichments.

*Comparing expression of genes in minimal medium with LDPE versus glucose culture*

We used Prodigal v2.6.3 (10) to annotate and predict genes from each sample's assembled contigs. EggNOG-mapper2 was employed to annotate the predicted genes against the EggNOG-v5 database (11), generating gene annotations based on KO, EC, and COG terms. We analyzed only genes with a total expression abundance ≥ 50 transcripts per million (TPM) for each gene.

For each gene, we calculated the relative transcript abundance by pooling all reads mapped to that gene across species and then computing reads per million (RPM) and TPM values (12, 13). We then calculated each gene’s average relative transcript abundance across the three replicates.

*Identifying and characterizing putative plastizymes*

To comprehensively investigate the genetic basis of plastic biodegradation, we developed a multi-step analytical approach that identifies known plastizymes and discovers potential new ones. This method combines database matching, expression data analysis, functional annotations, and statistical testing to provide a robust identification of genes likely involved in plastic degradation. We began with our metatranscriptomic dataset of 7,184 genes/proteins from cultures grown on LDPE or glucose. First, we identified known plastizymes by matching our gene IDs with those listed in the Plastic Microbial Biodegradation Database (PMBD: <http://pmbd.genome-mining.cn/gene/1/>; (14)) and Plastic Database (PDB: <https://plasticdb.org/>; (15)). In this first step, we identified 51 known plastizymes in our dataset. We conducted a paired t-test to determine whether the set of known plastizymes (as a whole) were significantly more highly expressed in LDPE than in glucose culture.

Next, we filtered the remaining genes for those with substantial expression (abundance ≥ 50 TPM) in the pool of LDPE and glucose conditions, resulting in a refined set of 4,969 transcripts (set N). We then divided this set into two complementary subsets: genes with higher expression in LDPE than in glucose cultures (T_L_ > 1.5T_G_, subset M, 1,027 transcripts) and those with lower or similar expression (T_L_ ≤ 1.5T_G_, subset M', 3,942 transcripts).

We categorized genes based on their functional annotations to identify putative plastizymes within subset M (T_L_ > 1.5T_G_, 1,027 transcripts). We considered a gene in subset M as a known plastizyme (10 transcripts) if it was found in the plastic databases as described above; we considered a gene in subset M as a putative plastizyme if it was not a known plastizyme but fell into one of several functional categories associated with plastizyme activity — oxygenases, dehydrogenases, hydrolases, lipases, esterases, and hydroxylases (Table S1). This subset K of putative plastizymes contained 182 genes from M.

We used a contingency test to determine whether the genes expressed substantially more (1.5X) in LDPE than in glucose tended to fall in the functional categories associated with plastizyme activity. This test excluded the known plastizymes. Additionally, we calculated odds ratios (equation 1) to quantify the enrichment of these putative plastizymes in LDPE conditions. This statistical analysis provided a measure of the strength of association between LDPE enrichment and putative plastizyme function.

$\text{Odds Ratio=}\frac{\text{K×((N-M)-K'))}}{\text{((M-K)×K')}}$ equation 1

Where: N = 4,969 (total number of genes with substantial expression), M = 1,027 (number of genes in the LDPE-enriched subset), K = 182 (number of enzymes in the LDPE-enriched subset that fell in the plastizyme functional categories of Table 1), K' = 387 (number of enzymes in the non-enriched subset that fell plastizyme functional categories).

*Statistical analysis*

All experiments were conducted in triplicate for each condition. Outcomes are expressed as the mean expression values ± standard deviation. We performed statistical tests using Microsoft Excel and R statistical software (version 4.3.1) (16). We conducted a Chi-square test to compare the phylum distributions between LDPE and glucose conditions. To assess the enrichment of known and putative plastizymes overrepresented in the LDPE condition, we performed Fisher's exact test. We calculated the odds ratio to quantify the magnitude of enrichment. We used a paired t-test to compare the overall plastizyme abundance in LDPE to that in glucose conditions. We applied the Bonferroni adjusted p-values < 0.05 to identify differentially enriched individual species between LDPE versus glucose culture.

**Supplementary Results**

*Visual evaluation of polyethylene-enriched Winogradsky systems*

Our Winogradsky column approach demonstrated the power of extended enrichment in fostering the development of stratified microbial communities. After 16 months, we observed a striking transformation from uniform layers to a vibrant array of colors, ranging from brown liquid at the aerobic top to light and dark green soils in anoxic zones (Figure S1). This visible differentiation aligned with previous findings (2, 17), illustrating our ability to establish niche-specific bacterial populations through the oxygen gradient. The distinct color patterns that developed over the 16-month incubation period served as a vivid reflection of the complex microbial niches and specialized populations that had been established.

*Transcription of genes enriched in glucose culture.*

Culture in glucose (without LDPE) enriched transcription of some genes for cell growth, sporulation, quorum sensing, and competence development, suggesting that microbial communities in the glucose culture were likely responding to nutrient deprivation. This is consistent with findings by (18) on survival mechanisms triggered by nutrient limitation. The differential expression of these genes highlights the distinct metabolic strategies employed by microorganisms when faced with different carbon sources. In the glucose culture, the rapid depletion of this readily accessible carbon source likely triggered stress responses and adaptive mechanisms for survival. Conversely, the absence of these stress-related gene expressions in the LDPE culture suggests that microorganisms may be maintaining a more stable metabolic state, possibly due to the slow but consistent release of carbon from LDPE degradation. This observation aligns with (19), who noted that microbial communities adapted to recalcitrant carbon sources often exhibit different growth patterns and metabolic profiles compared to those grown on labile substrates.


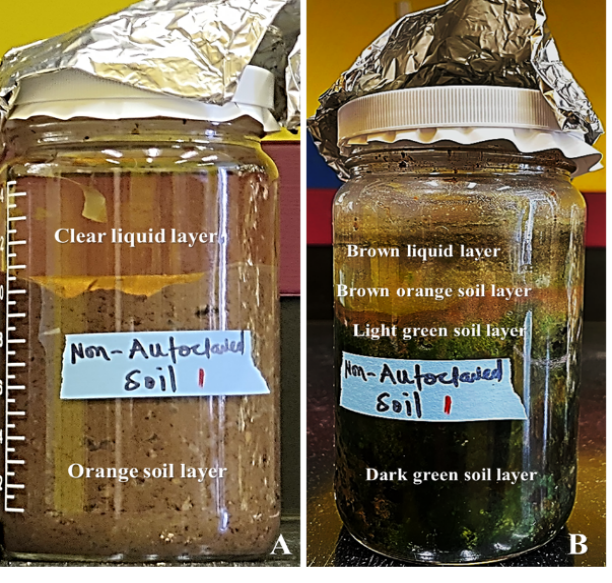


Figure S1. Modified Winogradsky columns enriched with PE strips. A.) Day 1 B.) Day 496. The column was prepared from farm soil. Plastic strips were placed at each layer with an inorganic Bushnell-Haas broth added to saturate the soil and to create an aqueous layer at the top.


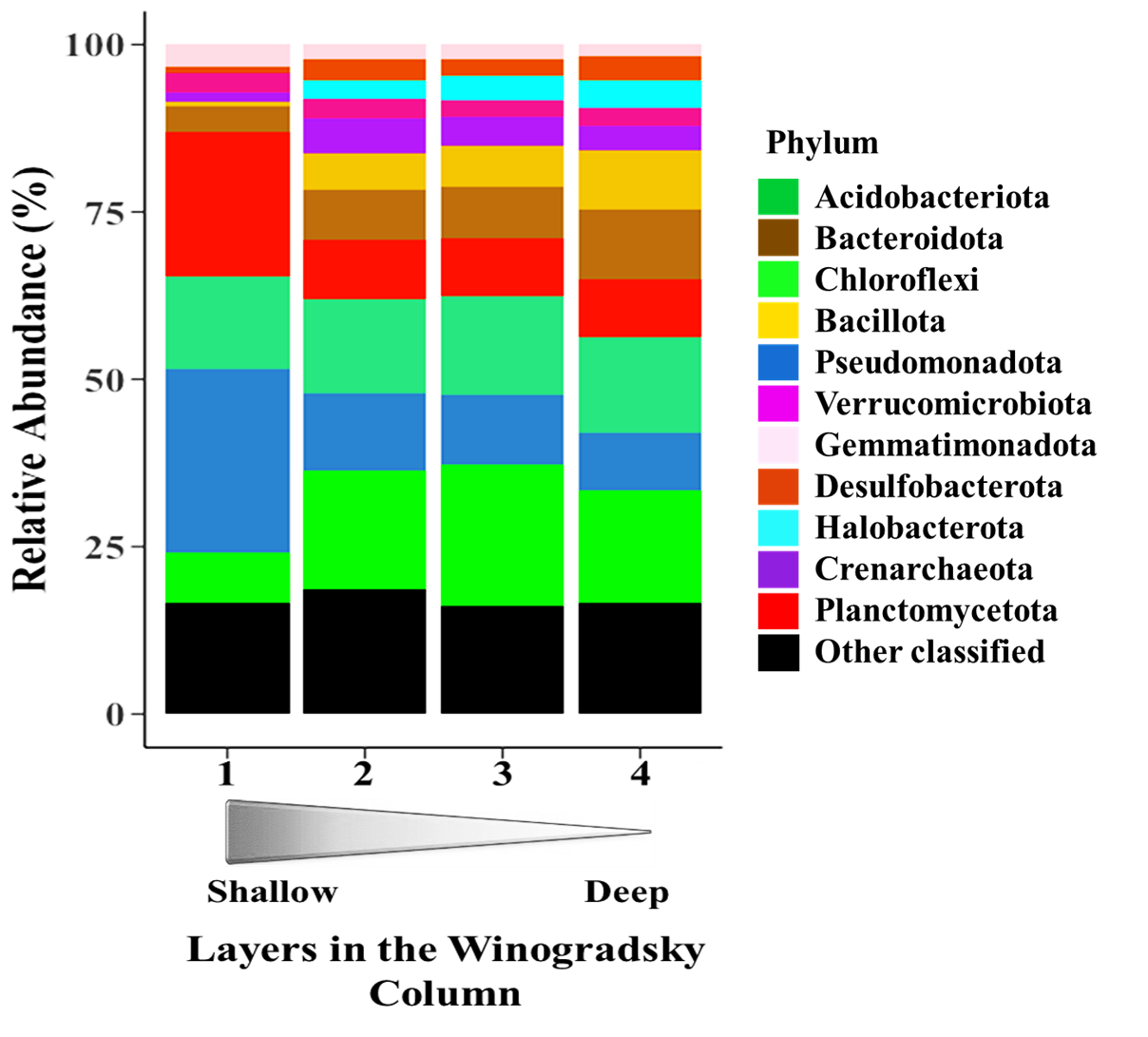


Figure S2. Relative abundance of top ten most-enriched bacterial phyla and other classified phyla in each layer of the modified Winogradsky column based on 16S rRNA survey. This figure represent phyla distribution of all-layers before enrichment in glucose solution versus LDPE powder.


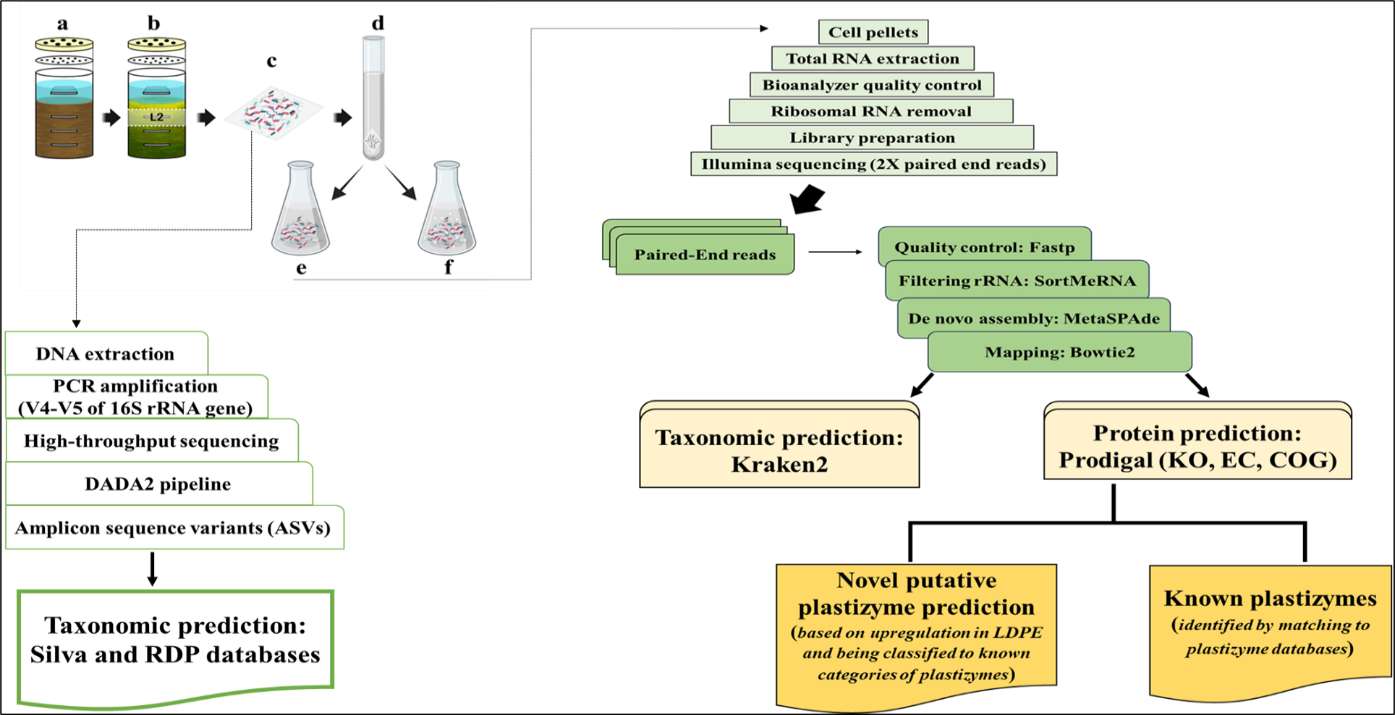


Figure S3. Experimental workflow and bioinformatic analyses. (a) Modified Winogradsky column at day 1. (b) Column after 487 days with stratified layers. (c) Layer 2 plastic strip harboring plastic-degrading microbes. (d) Vortexed to detach biofilms in 125 mL Bushnell Haas (BH) broth. For metatranscriptome analysis, the detached biofilms were used to inoculate (e) BH broth with 50 g/L glucose solution and (f) BH broth with 50 mg low-density polyethylene microplastic. Each experiment consisted of three replicates. Bioinformatic workflow includes DADA2 for 16S rRNA gene analysis and metatranscriptomic pipelines for functional gene analysis.

Table S1. Overview of enzyme classes involved in plastic biodegradation.

| Enzyme classes | Examples of known plastizymes in the plastic database | Phyla associated | References |
| --- | --- | --- | --- |
| Chitinase | chitin deacetylase; chitinase | Ascomycota, Mucromycota | (20, 21) |
| Catalase | catalase; catalase peroxidase | Ascomycota | (22) |
| Depolymerase | poly(3-hydroxybutyrate) depolymerase; polyhydroxybutyrate depolymerase | Pseudomonadota, Bacillota | (23, 24) |
| Dehydrogenase | aldehyde dehydrogenase; alcohol dehydrogenase | Pseudomonadota | (25, 26) |
| Hydroxylases | alkane hydroxylase; cytochrome P450 monooxygenase | Pseudomonadota, Ascomycota | (27, 28) |
| Hydrolases | mono (ethylene terephthalate) hydrolase; PET hydrolase; serine hydrolase | Pseudomonadota | (29, 30) |
| Esterase | carboxylesterase; esterase; para-nitrobenzyl esterase | Pseudomonadota, Bacillota | (31-33) |
| Oxygenase | ring-cleaving dioxygenase; biphenyl 2,3-dioxygenase subunit alpha | Pseudomonadota | (34, 35) |
| Lipase | triacylglycerol lipase; extracellular lipase | Actinomycetota | (30, 36) |
| Protease | protease; protease III | Actinomycetota, Bacillota | (37-39) |
| Oxidoreductase | oxidoreducatase family; phthalate | Bacillota; Pseudomonadota | (40, 41) |

a Enzyme classifications based on EC number system and molecular function

b Data compiled from PMDB (Plastic Microbial Database) and PlasticDB

c Phyla information represents documented microbial sources of each enzyme class

d References cited include experimental validation of plastic degradation activity

e Gene/protein names follow standard nomenclature from UniProt database

**Supplementary References**

1. Long Lane Farm. Long Lane Farm Annual Report 2019. 2020.

2. Olabemiwo FA, Kunney C, Hsu R, De Palo C, Bashaw T, Kraut K, et al. Searching for bacterial plastitrophs in modified Winogradsky columns. Front Microb. 2024;3.

3. Fadeev E, Cardozo-Mino M, Rapp J, Bienhold C, Salter I, Carvalho V, et al. Comparison of Two 16S rRNA Primers (V3–V4 and V4–V5) for Studies of Arctic Microbial Communities. Front Microbiol. 2021;12.

4. Callahan BJ, McMurdie PJ, Rosen MJ, Han AW, Johnson AJA, Holmes SP. DADA2: High-resolution sample inference from Illumina amplicon data. Nat Meth. 2016;13(7):581-3.

5. Weber N, Liou D, Dommer J, MacMenamin P, Quiñones M, Misner I, et al. Nephele: a cloud platform for simplified, standardized and reproducible microbiome data analysis. Bioinform. 2017;34(8):1411-3.

6. Shifu C, Yanqing Z, Yaru C, Jia G: an ultra-fast all-in-one FASTQ preprocessor. bioRxiv. 2018:274100.

7. Loman NJ, Misra RV, Dallman TJ, Constantinidou C, Gharbia SE, Wain J, et al. Performance comparison of benchtop high-throughput sequencing platforms. Nat Biotechnol. 2012;30(5):434-9.

8. Bankevich A, Nurk S, Antipov D, Gurevich AA, Dvorkin M, Kulikov AS, et al. SPAdes: a new genome assembly algorithm and its applications to single-cell sequencing. J Comput Biol. 2012;19(5):455-77.

9. Wood DE, Salzberg SL. Kraken: ultrafast metagenomic sequence classification using exact alignments. Gen Biol. 2014;15(3):R46.

10. Hyatt D, Chen G-L, LoCascio PF, Land ML, Larimer FW, Hauser LJ. Prodigal: prokaryotic gene recognition and translation initiation site identification. BMC Bioinform. 2010;11(1):119.

11. Cantalapiedra CP, Hernández-Plaza A, Letunic I, Bork P, Huerta-Cepas J. eggNOG-mapper v2: Functional Annotation, Orthology Assignments, and Domain Prediction at the Metagenomic Scale. Mol Biol Evol. 2021;38(12):5825-9.

12. Conesa A, Madrigal P, Tarazona S, Gomez-Cabrero D, Cervera A, McPherson A, et al. A survey of best practices for RNA-seq data analysis. Gen Biol. 2016;17(1):13.

13. Wagner GP, Kin K, Lynch VJ. Measurement of mRNA abundance using RNA-seq data: RPKM measure is inconsistent among samples. Theory Biosci. 2012;131(4):281-5.

14. Gan Z, Zhang H. PMBD: a Comprehensive Plastics Microbial Biodegradation Database. Database (Oxford). 2019;2019.

15. Gambarini V, Pantos O, Kingsbury JM, Weaver L, Handley KM, Lear G. PlasticDB: a database of microorganisms and proteins linked to plastic biodegradation. Database. 2022;2022.

16. R Core Team. R: A language and environment for statistical computing. R Foundation for Statistical Computing, Vienna, Austria. URL <https://www.R-project.org/>. 2020.

17. Esteban DJ, Hysa B, Bartow-McKenney C. Temporal and Spatial Distribution of the Microbial Community of Winogradsky Columns. PLOS ONE. 2015;10(8):e0134588.

18. Beskrovnaya P, Sexton DL, Golmohammadzadeh M, Hashimi A, Tocheva EI. Structural, Metabolic and Evolutionary Comparison of Bacterial Endospore and Exospore Formation. Front Microbiol. 2021;12.

19. Wilkes RA, Aristilde L. Degradation and metabolism of synthetic plastics and associated products by *Pseudomonas sp*.: capabilities and challenges. J Appl Microbiol. 2017;123(3):582-93.

20. Seenivasagan R, Karthika A, Poonkuzhali K. In vitro and in silico study of the efficacy of fungi in low-density polyethylene degradation in a disposal paper cup. W, A, & S Poll. 2022;233(3):77.

21. Urbanek AK, Arroyo M, de la Mata I, Mirończuk AM. Identification of novel extracellular putative chitinase and hydrolase from *Geomyces sp*. B10I with the biodegradation activity towards polyesters. AMB Expr. 2022;12(1):12.

22. Bhardwaj H, Gupta R, Tiwari A. Communities of Microbial Enzymes Associated with Biodegradation of Plastics. J Poly Env. 2013;21(2):575-9.

23. Martínez-Tobón DI, Gul M, Elias AL, Sauvageau D. Polyhydroxybutyrate (PHB) biodegradation using bacterial strains with demonstrated and predicted PHB depolymerase activity. Appl Microbiol Biotechnol. 2018;102(18):8049-67.

24. Wang YL, Lin YT, Chen CL, Shaw GC, Liaw SH. Crystallization and preliminary crystallographic analysis of poly(3-hydroxybutyrate) depolymerase from *Bacillus thuringiensis*. Acta Crystallogr F Struct Biol Commun. 2014;70(Pt 10):1421-3.

25. Ohta T, Tani A, Kimbara K, Kawai F. A novel nicotinoprotein aldehyde dehydrogenase involved in polyethylene glycol degradation. Appl Microbiol Biotechnol. 2005;68(5):639-46.

26. Kawai F, Hu X. Biochemistry of microbial polyvinyl alcohol degradation. Appl Microbiol Biotechnol. 2009;84(2):227-37.

27. Yoon MG, Jeon HJ, Kim MN. Biodegradation of polyethylene by a soil bacterium and AlkB cloned recombinant cell. J Bioremed Biodegrad. 2012;3(4):1-8.

28. Tseng W-S, Lee M-J, Wu J-A, Kuo S-L, Chang S-L, Huang S-J, et al. Poly(butylene adipate-co-terephthalate) biodegradation by *Purpureocillium lilacinum* strain BA1S. Appl Microbiol Biotechnol. 2023;107(19):6057-70.

29. Yoshida S, Hiraga K, Takehana T, Taniguchi I, Yamaji H, Maeda Y, et al. A bacterium that degrades and assimilates poly(ethylene terephthalate). Sci. 2016;351(6278):1196-9.

30. Danso D, Chow J, Streit WR. Plastics: Environmental and Biotechnological Perspectives on Microbial Degradation. Appl Env Microbiol. 2019;85(19):e01095-19.

31. Ribitsch D, Heumann S, Trotscha E, Herrero Acero E, Greimel K, Leber R, et al. Hydrolysis of polyethyleneterephthalate by p‐nitrobenzylesterase from *Bacillus subtilis*. Biotechnol prog. 2011;27(4):951-60.

32. Müller CA, Perz V, Provasnek C, Quartinello F, Guebitz GM, Berg G. Discovery of polyesterases from moss-associated microorganisms. Appl Env Microbiol. 2017;83(4):e02641-16.

33. Edwards S, León-Zayas R, Ditter R, Laster H, Sheehan G, Anderson O, et al. Microbial consortia and mixed plastic waste: pangenomic analysis reveals potential for degradation of multiple plastic types via previously identified PET degrading bacteria. I J Mole Sci. 2022;23(10):5612.

34. Tsuchii A, Takeda K. Rubber-degrading enzyme from a bacterial culture. Appl Env Microbioil. 1990;56(1):269-74.

35. Kasai D, Imai S, Asano S, Tabata M, Iijima S, Kamimura N, et al. Identification of natural rubber degradation gene in Rhizobacter gummiphilus NS21. Biosci, Biotechnol, and Biochem. 2017;81(3):614-20.

36. Mohanan N, Wong MC-H, Budisa N, Levin DB. Polymer-Degrading Enzymes of *Pseudomonas chloroaphis* PA23 Display Broad Substrate Preferences. I J Mole Sci. 2023;24(5):4501.

37. Li F, Wang S, Liu W, Chen G. Purification and characterization of poly (L-lactic acid)-degrading enzymes from *Amycolatopsis orientalis* ssp*. orientalis*. FEMS microbiol lett. 2008;282(1):52-8.

38. Oda Y, Yonetsu A, Urakami T, Tonomura K. Degradation of polylactide by commercial proteases. J of Poly Env. 2000;8:29-32.

39. Jahanshahi DA, Ariaeenejad S, Kavousi K. A metagenomic catalog for exploring the plastizymes landscape covering taxa, genes, and proteins. Sci Rep. 2023;13(1):16029.

40. Gui Z, Liu G, Liu X, Cai R, Liu R, Sun C. A deep-sea bacterium is capable of degrading polyurethane. Microbiol Spectr. 2023;11(3):e00073-23.

41. Li D, Yan J, Wang L, Zhang Y, Liu D, Geng H, et al. Characterization of the phthalate acid catabolic gene cluster in phthalate acid esters transforming bacterium-*Gordonia sp*. strain HS-NH1. I Biodeterior & Biodegrad. 2016;106:34-40.
